# Not all “distractor” tags are created equal: using a search asymmetry to dissociate the inter-trial effects caused by different forms of distractors

**DOI:** 10.3389/fpsyg.2014.00669

**Published:** 2014-06-30

**Authors:** Alejandro Lleras, Simona Buetti

**Affiliations:** Department of Psychology, University of Illinois at Urbana-Champaign, ChampaignIL, USA

**Keywords:** attention, pop-out, search asymmetry, distractor effects, trial history, inter-trial effects

## Abstract

In a typical pop-out task, there is one target and a varying number of distractor stimuli. Now imagine a target-absent display in the context of a pop-out task: all items are identical, and it is decidedly easy to conclude that all items in the display are distractors, precisely because there is no target to select on that display. One may be tempted to say that, as far as the attention system is concerned, these two types of distractors are the same: target-present distractors and target-absent distractors. The present study proposes that this is actually not the case. Target-absent distractors can sometimes produce inter-trial effects that their close-cousins, the target-present distractors, cannot. We used a letters/numbers categorical oddball task to demonstrate this difference. The results are interpreted in the context of recent findings in cognitive neuroscience as well as cognitive modeling.

## INTRODUCTION

There has been substantial interest in studying inter-trial effects in visual search experiments in which there is uncertainty as to what the target might be on the next trial. For example, in oddball experiments, participants must quickly decide which item is the visual oddball and then make a response to it, such as saccade toward it (e.g., Becker, 2008; Caddigan and Lleras, 2010; Tseng et al., 2014), or respond to some attribute of the oddball that requires the target be scrutinized to some degree (e.g., Maljkovic and Nakayama, 1994; Ariga and Kawahara, 2004; Goolsby et al., 2005; Kristjánsson and Driver, 2008; Lamy et al., 2008; Lleras et al., 2008). In this literature^1^, most often, there are two categories of stimuli (e.g., Maljkovic and Nakayama, 1994; Ariga and Kawahara, 2004), though sometimes three (e.g., Lleras et al., 2008) or four (e.g., Lamy et al., 2008) from which oddball and distractors are picked. The categories are sufficiently visually distinct from each other as to give rise to the phenomenon of pop-out. When set size is manipulated, reaction time (RT) to find the target is either not influenced by set size (e.g., Wan and Lleras, 2010) or sometimes decreases with increasing set size (e.g., Meeter and Olivers, 2006). Some of the initial interest in these oddball search inter-trial effects came from the following apparent contradiction: if the oddball is a pop-out (and by that we understood a stimulus with sufficient bottom-up salience as to capture attention automatically onto itself), why should there be *any* inter-trial effects in an oddball task at all? How can an automatic pre-attentive computation, such as the detection and automatic orienting toward of a pop-out, be improved upon by simple repetition?

One hint that the answer to this question lies outside of the domain of automatic, perceptual processing types of mechanisms is the simple observation that no such inter-trial effects are ever found when the task is not to orient to the target but simply to *detect* the presence or absence of the oddball (e.g., Lleras et al., 2008). That is, if these inter-trial effects arose from changes in the manner in which features are perceived, or encoded or even represented at early stages of visual processing, then one would have to predict that inter-trial effects would be observed irrespective of the task that participants *perform* on the stimulus. If seeing, for example, a red target amongst green distractors on trial N-1 facilitates the encoding of red items on trial N, then one would expect that the detection of a red oddball on trial N should be facilitated, yet it is not. It appears then that the priming effect is not an advantage in detecting and reporting the presence of a specific visual segmentation in the display (homogeneous display vs. not) neither a matter of preferential encoding. Rather it arises from the need to *orient* to one of the items in the display (the oddball). The rub, though, is that participants donot know which feature will determine target status on any given trial. Inter-trial priming in this pop-out oddball tasks, thus, seems then unrelated to the visual conspicuity of the target. In fact, the same magnitude priming effect is found when the two visual categories used in the task are very different from each other (red vs. green) than when they are very similar to each other (equiluminant fuschia and pink colors), in spite of the fact that differences in feature discriminability produce large changes in overall RT, with easier to discriminate pairs producing faster RTs (Wan and Lleras, 2010).

Two different types of inter-trial effects in oddball search tasks have been documented: the so-called “priming of pop-out” effect (PoP, Maljkovic and Nakayama, 1994), and the “distractor-previewed effect” (DPE, Goolsby et al., 2005). PoP is a reduction in RTs when the oddball on a given trial is repeated on the following trial. For instance, responses to a red-amongst-green oddball on trial N will be faster if the oddball on trial N-1 had also been red. Note that in PoP, there can also be repetition (or not) of distractor stimuli across trials. In the previous example, that would occur if on trial N-1 the distractors had been green. Such repetitions can yield further reductions in RT (Kristjánsson and Driver, 2008; Lamy et al., 2008). The DPE is measured in experiments much like those measuring PoP with the difference that target-absent trials (i.e., displays where items are all identical) are included in the series of events. For instance, responses to a red target will be slower on trial N if trial N-1 had been a target-absent trial in which all items were red, than when those elements were of a different color (say green). This effect is observed in both spatial and temporal [Rapid serial visual presentation (RSVP), measured as a drop in accuracy] search tasks, and also, when both types of search tasks are interleaved (Lleras et al., 2009a,b). Finally, it is worth noting that, compared to a neutral baseline where no features repeat between trials, a DPE is composed of: (1) a difficulty in orienting to a target recently associated with distractor status (e.g., a target-absent diplay AAA followed by a target-present display ABB is slower than a display absent display CCC followed byABB), and (2) a small but reliable advantage at rejecting anew distractors that were recently rejected (BBB- > ABB is faster than CCC- > ABB, see Lleras et al., 2008).

It has been argued that one methodological advantage in studying the DPE is that it is an effect associated with the change in status of a single feature (e.g., Lleras et al., 2009a; Scalf et al., in press). That is, because only one relevant feature can repeat from a target-absent trial to a target-present trial (the feature shared by all distractors in a target-absent trial), the effect is focused on that single feature. Also, repetitions of irrelevant features also do not produce a DPE: if the task is to report a color oddball, seeing a display with items of identical shapes does not increase RT on a subsequent trials for target of that same shape (Levinthal and Lleras, 2008a). Thus, the DPE is a very focused and easily tractable effect whereas PoP tends to be a more complicated phenomenon: because more features can jointly impact performance across trials (e.g., Michal et al., submitted; Huang et al., 2004; Kristjánsson and Driver, 2008; Lamy et al., 2008). Somewhat surprisingly, the DPE effect has been observed in several experiments in which the corresponding PoP was not observed (Ariga and Kawahara, 2004). This is somewhat puzzling because if priming in pop-out search can be influenced both by repetitions of target and distractor status across trials, one would imagine that it would be more easily observed simply because there are more underlying sources of priming. It is all the more puzzling when one considers that this single dissociation is observed in experiments with only two feature categories. Say the categories are A and B. In the critical DPE sequence, we would have a target-absent trial (for instance a display containing AAA) followed by a target-present trial (ABB). This would produce a measurable difficulty (slowdown) in responding to the target (A) on the second trial, because A has undergone a status change from distractor (on trial N-1) to target (on trial N). Now consider the corresponding PoP sequence. We have a target B surrounded by As on trial N-1 (BAA), followed by a display where A is surrounded by Bs (ABB). As far as the history of A is concerned, the DPE and PoP sequences are identical: in both cases A was tagged as distractor on trial N-1 and was tagged as target on trial N. Yet, on the DPE sequence of trials (target-absent to target-present) an inter-trial effect is observed, whereas, for certain visual categories (e.g., male/female oddball task and motion direction oddball task), the corresponding PoP is not. We ask the question: why is this the case?

The goal of the current paper is to provide a tentative first explanation for this asymmetry. The difference, we propose, is on the emphasis of what is being actively tagged in both scenarios, which reflects the different state of uncertainty in the two tasks. On DPE sequences, there are trials where participants do not respond, whereas on PoP sequences, participants respond on every trial. If the task in an experiment is to select oddballs, target/oddball-absent displays represent a form of “failure” in the human–environment interaction, because the environment is not allowing participants to perform the stated goal (Lleras et al., 2009a). As a result, in a target-absent trial the emphasis is placed on categorizing the feature with a “distractor” tag, which is associated with the “no-go” response^2^. Importantly, this assignment occurs in the absence of other visual features. In contrast, on target-present trials, the emphasis, we argue, is mostly on the relative success experienced on the current trial (i.e., the act to select, when the goal is to select). That is, because an easy-to-select oddball is present, the emphasis is placed on tagging the oddball’s feature as “target.” Importantly, the tagging of the distractor feature (not unimportant in magnitude, see Lamy et al., 2008) we think comes as a *consequence* of the tagging of the target feature. Thus, in a display containing ABB, *because* A is easily selected and tagged as target, B is then tagged as distractor. To account for the single dissociation between the DPE and the PoP, we suggest that, in PoP, because distractor tagging is dependent on target tagging, visual categories that donot allow for easy target tagging *cannot* produce a PoP. In other words, if the target does not pop-out, the target feature will not be tagged, and as a consequence, neither will the distractor feature. Therefore, there will be neither a target tag nor a distractor tag in place to influence performance on the subsequent trial. In other words, no PoP can be observed. In contrast, in the DPE, distractor tagging on target-absent trials occurs in the absence of target tagging. It is an integral part of terminating attentional engagement on the trial. Therefore, this distractor tag is put in place on target-absent trials and can then impact the allocation of attention in the subsequent trial, resulting on a measurable inter-trial priming effect (i.e, the DPE).

To test our differential-tagging hypothesis, that is, that target-present distractor tags are not the same as target-absent distractor tags, we compared performance on PoP and DPE with visual categories that are relatively similar in terms of low-level features. This allowed us to equate possible confounds with low-level differences between categories that do or do not produce PoP. It had the added benefit that these two categories can sometimes produce pop-out effects and sometimes not: a letter may pop-out amongst numbers, whereas the reverse may not be true (e.g., Brand, 1971; Jonides and Gleitman, 1972). With the stimuli and configurations we used, we found a search asymmetry: letters popped-out amongst numbers but the reverse was not true (Experiment 1). The presence of this asymmetry allowed us to demonstrate, without changing the task or the stimuli, a fundamental difference between tags obtained in target-absent displays and those obtained in target-present displays. Specifically, if tags on target-present trials require the presence of a target, then we would predict that only when a target pops out can PoP be observed. That is, we expected to find significant PoP on trials where the target was a letter but not on trials where the target was a number. In contrast, in the DPE, the assignment of distractor tag does not depend on the pop-out qualities of that stimulus because it is defined on target-absent trials (where pop-out is not happening). Thus, we expected to find DPEs both when letters were targets and when numbers were targets. Moreover, because letters pop-out amongst numbers, we also expected faster overall RTs on those trials, than on trials with number targets. All of these predictions were confirmed.

It is necessary to mention that the letter/number categorical effects are not always observed (e.g., Duncan, 1983; Krueger, 1984). That said, we were simply interested in the fact that, given that we had obtained one with our specific set of stimuli, then this asymmetry permits us to test our hypothesis of differential-tagging. Experiment 1 documented the presence of the search asymmetry with our letter/number stimuli, whereas Experiments 2 and 3 were designed to measure PoP and DPE, respectively, with those stimuli.

Finally, an additional interest in using numbers and letters as stimuli in the search task was to add further evidence to the categorical nature of the PoP and DPE effects. Lleras et al. (2009b) have already demonstrated that a temporal DPE (using temporal search through RSVP streams) could be observed using letters and numbers as categories, but these categories have never been used in PoP studies. Using such complex and visually similar categories to define oddball status is interesting and theoretically valuable because it allows us to minimize to the largest extent possible sources of inter-trial priming arising from modulations of low-level feature processing (so-called feature gain modulations, as proposed by some authors Wolfe et al., 2003; Lee et al., 2009).

## GENERAL METHODS

### PARTICIPANTS

Participants completed the experiments in exchange for course credit in a psychology course. Eighteen completed Experiment 1, and twenty subjects each completed Experiments 2 and 3. We used an inclusion criterion of 90% overall correct responses. This accuracy cut-off reduced our sample to 15, 19, and 14 subjects, respectively.

### APPARATUS AND STIMULI

**FIGURE 1:**
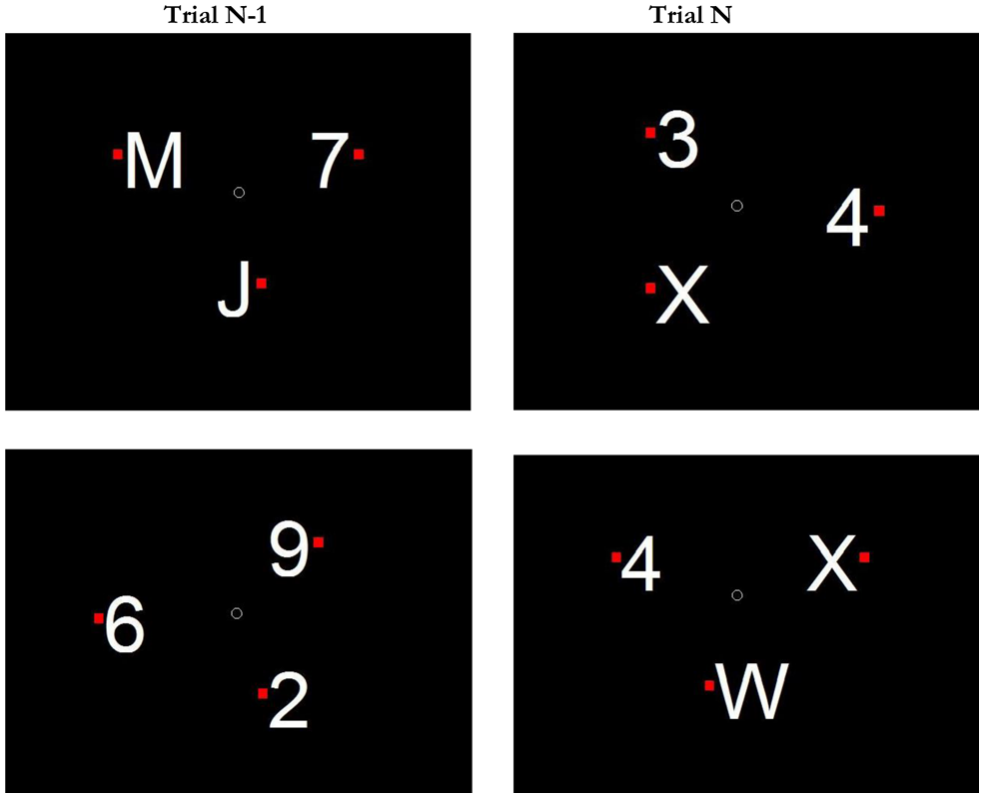
**Schematic illustration of stimuli and conditions in Experiments 2 and 3.** Top Row: example of a Switch Search condition on trial N. The category-oddball is a letter (X). Participants must respond Left, because the X’s red dot is to the left of the X. Note that on trial N-1, letters were associated with distractor status (the target was a number). Bottom Row: example of a Target-Category Previewed Condition on trial N. The category oddball is a number (4). On trial N-1, numbers were associated with distractor status.

Stimuli were presented on a 17-inch CRT monitors, controlled by a Dell Optiplex PCs, using the Psychophysics Toolbox (Brainard, 1997; Pelli, 1997). Stimuli could be letters (picked from the set C D J K M R UW X) or single-digit numbers (all but zero) in Arial font, ∼1.89^∘^ of visual angle tall. In Experiment 1, set-size was manipulated, so there could be either 3, 4, or 6 items on a given display. In Experiments 2 and 3 displays always had three equally spaced items. Items were always presented along an iso-acuity ellipse (Rovamo and Virsu, 1979) of ∼9.5^∘^ of visual angle across the horizontal axis and 8^∘^ across the vertical axis. To the right or left of each character, we presented a small red square (0.28^∘^ tall). See **Figure 1** for an illustration of a sample display. The search display contained a small dot in the center of the display and participants were encouraged to keep their eyes at fixation prior to the start of the trial.

## EXPERIMENT 1: LETTER-NUMBER SEARCH ASYMMETRY

The goal was to document the letter-number search asymmetry with our stimuli and displays.

### DESIGN AND PROCEDURE

There was an equal number of target-present (one oddball item) and target-absent trials (no oddball). The oddball was equally likely to be a letter or a number. And every set size was equally likely on every trial. There were 50 repetitions for every possible combination of set-size, target presence and oddball type condition.

Displays were presented until response. Participants were asked to press the up-arrow key to report the presence of an oddball and the down-arrow key to report the absence of one. There was a variable inter-trial interval between 0.8 and 1.3 s. A 2 s delay was imposed following incorrect responses during which the entire display was blank. Participants were told that the best strategy in the task was to keep their eyes at fixation, though eye-movements were not monitored.

### RESULTS

Average accuracy was very high (96.9%), but nonetheless appeared to vary by search condition, particularly in the number oddball condition at high set sizes. To correct for the possibility of speed-accuracy trade-offs in the search task, in addition to analyzing correct RTs, we also computed search efficiency scores by dividing RTs by the accuracy (in terms of proportion correct), for each condition, for each participant.

The goal of the analysis was to evaluate the magnitude of the search slope, on target-present trials with letter and number targets separately. To do so, we compared RTs between set sizes 6 and 3. First, we analyzed correct trials where letters were targets: there was no effect of set size on RTs, *F*(1,14) = 0.06, or on efficiency scores, *F*(1,14) = 0.14. The average search slope for letters amongst numbers was 3.6 ms/item. These results are consistent with the hypothesis that letters pop-out amongst numbers. The same analysis on trials where numbers were targets showed a marginal effect of set size on correct RTs, *F*(1,14) = 3.72, *p* = 0.074. The effect of set size on efficiency scores was significant, *F*(1,14) = 5.48, *p* = 0.035. The average search slope for numbers amongst letters was 14.4 ms/item, consistent with the hypothesis that with our stimuli, numbers did not pop-out amongst letters. Finally, we compared the magnitude of the search slopes against each other, but the comparisons did not reach significance in either RTs (difference = 10.7 ms/item, *t*(14) = 0.68, *p* > 0.05) nor efficiency score (difference = 14 ms/item, *t*(14) = 0.78, *p* > 0.05).

### DISCUSSION

The data from Experiment 1 showed that with our stimuli, we find a search asymmetry between letters and number stimuli. Letters clearly popped-out amongst numbers, whereas the converse was not true. We should add that the distinction that is critical for our argument is whether there is evidence for “guidance” in one search and not in the other. As guideline, we simply used the rule of thumb that a search is “efficient” (i.e., a pop-out or guided) when its search slope is zero or near-zero (in the 0–10 ms/item range), whereas it is considered to be inefficient when it is different from 0 and numerically larger than 10 ms/item (e.g., Wolfe and Horowitz, 2004).

## EXPERIMENT 2: PRIMING OF POP-OUT AND LETTER-NUMBER SEARCH ASYMMETRY

We used the same stimuli as in Experiment 1 in a priming of pop-out task to test our hypothesis. As a reminder, we had predicted that a PoP effect would be observed with letter targets but not with number targets because priming of pop-out requires that pop-out occurs in order to establish target and distractor tags. Priming of pop-out is computed as the difference in RT between two conditions: Switch Search RT (target-distractor assignment switched from trial N-1 to trial N) minus Repeat Search RT (target-distractor tags remained the same from trial N-1 to trial N). For example, the sequence LNN- > NLL is a Switch Search condition and the sequence NLL- > NLL is a Repeat Search condition. Note that on both cases, the RT is evaluated on an identical display (NLL).

### METHODS

The methods were identical to those of Experiment 1 except where noted.

### DESIGN AND PROCEDURE

All trials contained a category oddball. There were a total of 500 trials. No time penalty was imposed following incorrect trials. On average, there were 118 Repeat Search and 124 Switch Search trials with letters as targets and 130 Repeat Search and 124 Switch Search trials where numbers were targets. Participants were asked to find the oddball character in the display (letter amongst numbers or number amongst letters) and report the location of the red dot next to the oddball by pressing the left-arrow and right-arrow button, for left-side and right-side dots respectively. One additional constraint was imposed on the stimuli: to ensure that we were measuring inter-trial effects driven by categorical information, characters never repeated from one trial to the next.

### RESULTS

Average accuracy in this task was 95.4%. Not surprisingly, participants were more accurate on trials with letter targets (96.5%) than with number targets (94.8%), *F*(1,18) = 9.10, *p* = 0.007. Similarly, participants were more accurate on Repeat Search trials (96.2%) than on Switch Search trials (95.0%), *F*(1,18) = 7.67, *p* = 0.013. The interaction was not significant, *F*(1,18) = 0.037, *p* = 0.85. Given these systematic changes in accuracy by condition, we analyzed both correct RTs and efficiency scores in this task to evaluate the presence of a priming-of-pop-out.

**FIGURE 2:**
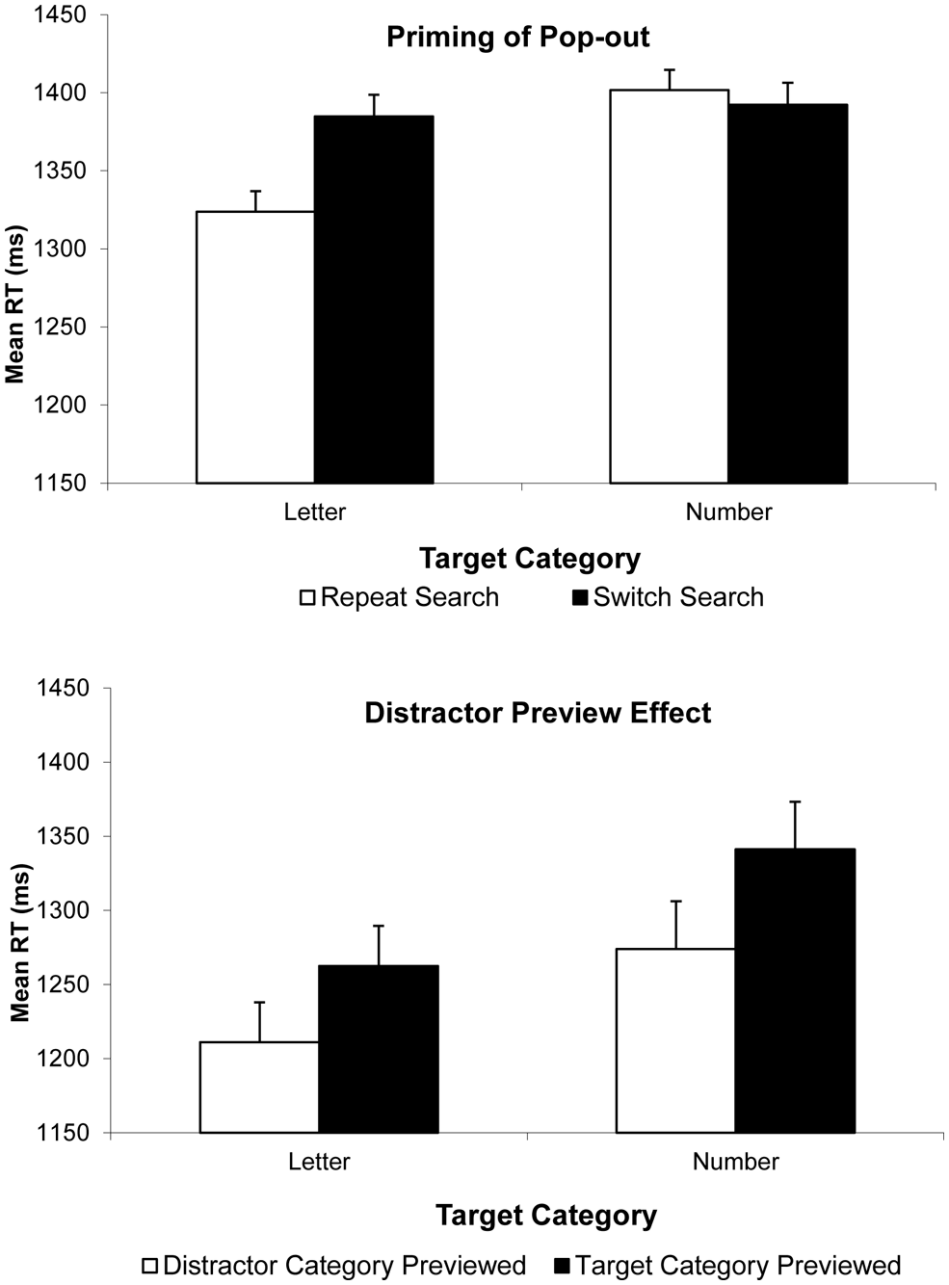
**Results of Experiments 2 (Top) and 3 (Bottom).** Correct mean RTs are plotted as a function of target type (letter or number) on the current trial and trial type: Repeat Search and Switch Search conditions, for PoP in Experiment 2; and Distractor-Category Previewed and Target-Category Previewed conditions, for DPE in Experiment 3. Error bars indicate the standard error of the mean.

**FIGURE 3:**
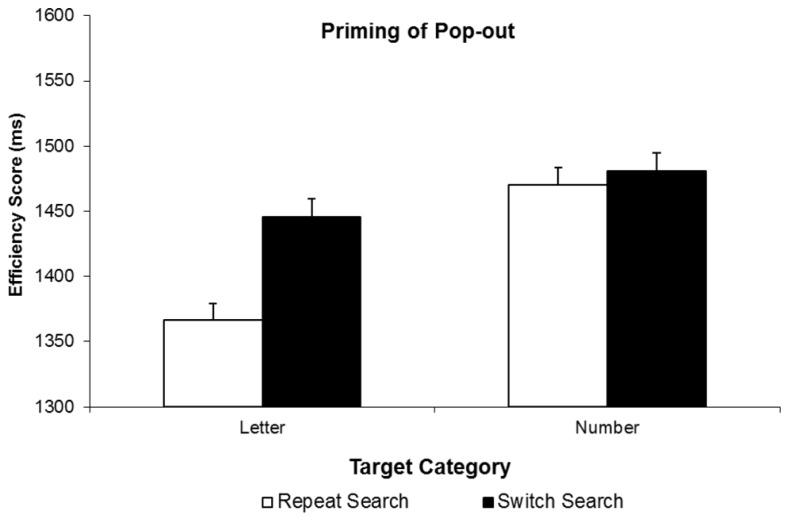
**Results of Experiment 2.** Efficiency scores are plotted as a function of target type (letter or number) on the current trial and trial type: Repeat Search and Switch Search conditions, for Priming of Pop-out. Error bars indicate the standard error of the mean.

We first submitted correct RTs to a two-way analysis of variance (ANOVA) with factors target type (letter vs. number) and search condition (switch vs. repeat). Overall, RTs were faster for letter targets than number targets, *F*(1,18) = 6.25, *p* = 0.022, and faster on Repeat Search than on Switch Search conditions, *F*(1,18) = 7.10, *p* = 0.016, but more crucially, these main effects were qualified by a significant interaction, *F*(1,18) = 11.49, *p* = 0.003. Planned follow-up tests revealed that in fact, there was a significant 61 ms PoP observed on trials with letter targets, *t*(18) = 4.54, *p* < 0.001, whereas there was no corresponding (-10 ms) PoP on trials where numbers were trials, *t*(18) = -0.62, *p* = 0.54. **Figure 2**, top, illustrates these findings. Most importantly, given the concerns about systematic changes in accuracy by search condition, we found an identical pattern of results where search efficiency scores were analyzed, with a significant type by search condition interaction, *F*(1,18) = 6.29, *p* = 0.022, with a significant 79 ms PoP when letters were targets (measured in efficiency score), *t*(18) = 4.47, *p* < 0.001, and an non-significant 11 ms PoP when numbers were targets, *t*(18) = 0.62, *p* = 0.541 (see **Figure 3**).

### DISCUSSION

The data from Experiment 2 confirmed our predictions: we found a clear and significant priming of pop-out effect on trials were letters were the target, but not on those where numbers were targets, parallels the findings of Experiment 1 in which we found that, with our stimuli and displays, letters pop-out amongst numbers while the converse is not true. The critical question now is whether we will find a similar asymmetry with these stimuli in the Distractor Preview Effect, or not.

## EXPERIMENT 3: DISTRACTOR PREVIEW EFFECT AND LETTER-NUMBER SEARCH ASYMMETRY

We used the same stimuli and procedures as in Experiment 2, except that we incorporated into the design target-absent trials, so that the DPE could be evaluated. As a reminder, we expected to find a DPE both on trials where a letter is the target and on trials where a number is the target. The Distractor Preview effect is computed as the difference in RT between two conditions: the target-category previewed condition RT (the target on trial N belongs to the category of items present in the preceding target-absent trial) minus the distractor-category previewed condition RT (the distractors on trial N belong to the same category as the distractors on the preceding target-absent trial). For example, the sequence NNN- > NLL is a target-category previewed condition, and the sequence LLL- > NLL is a distractor-category previewed condition. Again, the effect is measured on identical displays (performance on trial NLL).

### METHODS

Methods were identical to those of Experiment 2, except were indicated.

### DESIGN AND PROCEDURE

Trials with no category oddball were included in the trial sequence. To ensure sufficient trials were observed in each of the experimental conditions, and in agreement with previous DPE papers, 80% of trials were paired into target-absent followed by target-present trial sequences. The remaining 20% of trials were divided into 10% of target-absent followed by target-absent trials and 10% of target-present followed by target-present trials. The trial pairs were randomly shuﬄed for each subject to produce the sequence of events in the experiment. Of the 80% of trial pairs designed to evaluate the DPE, there was an equal amount of trials with letter targets and with number targets in the target-present trial, and there was an equal amount of trials with letter distractors in the target-absent trials as target-absent trials with number distractors. Finally, target-absent displays were presented only for 600 ms and participants were asked to withhold any responses on those trials.

### RESULTS

For entirely unanticipated reasons, many subjects had difficulty in this task and had to be excluded from the analysis. This may have been due to the introduction of target-absent displays (which require withholding of responses), but also, we suspect, to the time of year where the subjects were run (near the end of the semester). The accuracies of the rejected subjects was low sometimes spectacularly so, for such a relatively easy task (79, 78, 74, 54, 64, and 79%). In comparison, the overall average for the remaining subjects was 94.6%. Accuracy on DPE trials (target-present trials preceded by a target-absent display) was analyzed in a two-way ANOVA with factors target type (letter vs. number) and trial type (target-category previewed vs. distractor-category previewed). Accuracy was significantly better on trials with letter targets (94.8%), than on trials with number targets (91.2%), *F*(1,13) = 11.35, *p* = 0.005. Similarly, responses were more accurate on distractor-category previewed trials (94.0%) than on target-category previewed trials (92.0%), *F*(1,13) = 8.29, *p* = 0.013. The target-type by trial type interaction approached but did not reach significance, *F*(1,13) = 3.84, *p* = 0.072. Given these systematic changes in accuracy by condition, we analyzed both correct RTs and efficiency scores in this task to evaluate the presence of a DPE.

We first submitted correct RTs to a two-way ANOVA with target type and trial type as factors. RTs were faster when letters were targets than when numbers were targets, *F*(1,13) = 7.98, *p* = 0.014. Further, RTs were slower on target-category previewed trials than on distractor-category previewed trials, *F*(1,13) = 8.25, *p* = 0.013, and there was no hint of a target type by trial type interaction, *F*(1,13) = 0.18, *p* = 0.67. In other words, we obtained a significant DPE effect for both target types, and this effect was not modulated by trial type (see **Figure 2** bottom). That said, given the concerns about accuracy, we ran the same analysis on efficiency scores. These analyses corroborated the conclusions of the correct RT analysis. The target type by trial type interaction failed to reach significance, *F*(1,13) = 2.28, *p* = 0.155. But importantly, even if it had reached significance, our specific hypothesis was not that there would be no modulation by target category, but rather, that a significant DPE would be observed both when letters were targets and when numbers were targets. Planned follow-up analyses confirmed this. With letter targets, there was a 51 ms DPE in RT, *t*(13) = 2.07, *p* = 0.059, and a 66 ms effect measured in efficiency score, *t*(13) = 2.196, *p* = 0.047. More critically, and unlike what we observed in Experiment 2 in PoP, with number targets, there was a 66 ms DPE in RT, *t*(13) = 2.26, *p* = 0.042 and a 140 ms effect measured in efficiency score, *t*(13) = 2.67, *p* = 0.019.

### DISCUSSION

The results of Experiment 3 confirmed our hypothesis. First, we obtained a significant DPE on trials where the target was a letter. Presumably everyone would expect such a result, given that letters pop-out amongst numbers, and that a significant PoP effect was observed for this category of stimuli in Experiment 2. More crucially, on trials where the target was a number, we obtained significant inter-trial priming (a DPE) even though no corresponding inter-trial priming was observed when numbers were targets in Experiment 2 (no PoP). This pattern of results confirm the presence of a single dissociation between PoP and DPE, that suggests that there is something unique to the “distractor” tags that are put in place in the DPE, which is quite different from the distractor tags that are at play in PoP.

## GENERAL DISCUSSION

The goal of this paper was to examine one possible reason why, with the same stimuli, one may be able to observe a DPE, when the corresponding PoP is non-existent. This question is important because it speaks to the issue of target-distractor role assignment, which presumably drives many inter-trial effects in search, like PoP and the DPE. We proposed that the circumstances through which the tagging is done affects whether an inter-trial effect will be observed or not. The big contrast, of course, is that in PoP trials, when there is an obvious target (i.e., the pop-out), the presence of this item in the display drives the target-distractor tag assignments. That is, because a pop-out is found, whatever feature was responsible for defining that oddball gets quickly and easily tagged as target, and as a result of this tagging, the other feature present in the display is tagged as associated with distractor status. Thus, we proposed that when there is no pop-out on trial N-1, there can be no priming on trial N. In contrast, in the DPE the distractor tag that is activated on trial N-1 occurs not because of the presence of an oddball in the display, but because of its absence. The attention system fails to find a feature to select in the display (because there is none) and associates the feature that is visible with this absence of selection, i.e., a distractor tag. Because this tag exists, then it can modulate performance on trial N.

The data presented here was consistent with our differential-tagging hypothesis. Using the number/letter search-asymmetry, we showed that when a display produced a pop-out, an inter-trial effect was observed (i.e., when letters were targets), and when a display failed to produce a pop-out, no inter-trial effect was observed. Note that the advantage of using a search asymmetry is that we could see both types of search performance (pop-out and somewhat inefficient search) with the same stimuli, and thereby avoid confounds that would occur when analyzing PoP across very different types of stimuli (say comparing the presence of PoP with color pairs and the absence of PoP with male/female oddballs). In contrast, we did observe a reliable DPE with both types of target categories.

One further reason to believe that distractor tags on target-absent displays are different from distractor (and target) tags on target-present displays comes from a recent functional magnetic resonance imaging (fMRI) study by Scalf et al. (in press). In that study, the authors used a categorical oddball task with houses and faces as the two categories. Most critical, they found regions of the brain in what is traditionally considered to be the ventral attention system (VAS, such as the right middle frontal gyrus, inferior frontal gyrus, and right supramarginal gyrus) to be more active on target-category previewed trials than on distractor-category previewed trials. That is, evidence was consistent with the idea that the VAS was sensitive to distractor tags from a preceding target-absent trial. Specifically, this increased activation suggested that it was harder to direct attention toward the oddball on target-category previewed trials than on distractor-category previewed trials. In contrast, the one previous fMRI study of PoP (Kristjánsson et al., 2007, who used a color pop-out task) had found that the PoP was mostly observed in brain regions in the dorsal attention network (DAN) like the frontal eye fields (FEFs) and intraparietal sulcus (IPS). Thus, the simple dissociation that we observed here between PoP and DPE with number targets is consistent with the finding that there seems to be a neural dissociation between these two effects.

One question that our design cannot clearly address is whether the DPE observed on number-target trials is driven by a cost associated with the role-reversal of the number category (i.e., a slow-down in the target-category previewed condition) or by an advantage due to the repetition of a consistent role for the letter category (i.e., a facilitation on distractor-category previewed condition). Let’s compare the two conditions. The DPE when a number is a target is measured as faster RTs on the sequence LLL- > NLL than on the sequence NNN- > NLL. Therefore, the DPE could be due to the fact that, given that letters are relatively salient (they pop-out), that perhaps when letters are tagged as distractors (LLL) and they re-appear on the subsequent trial as distractors (NLL), that these stimuli will be more easily discarded as potential targets and attention will more easily move toward the number oddball, producing an RT advantage.

The alternative, of course, is that the effect is actually driven by the switch in role of the number category: when numbers are tagged as distractors (NNN), it may be all the more difficult to select a number on the subsequent trial (NLL), even if numbers are not particularly powerful pop-outs amongst letters. This alternative would be in keeping with the fact that, in general, most of the DPE is observed as a difficulty in orienting toward a recently tagged-as-distractor feature (e.g., Lleras et al., 2008, 2009b; Caddigan and Lleras, 2010). This account is also consistent with the fact that in Experiment 3, the slowest condition was the target-category previewed condition with number targets, as if participants had suffered both from numbers being harder to select, overall, and further, from an additional bias against selecting numbers. It is also consistent with the results of Experiment 2, which suggested that the effects obtained with these displays are driven by the history of the current target: only when a letter was repeated across consecutive trials was there an inter-trial effect (a benefit) observed.

What is clear from our data is that, in two-feature oddball tasks, for a DPE to be observed, it is sufficient that one of the two visual categories used in the task produces pop-out, whereas they suggest that for a PoP to be observed both categories must produce a pop-out. Thus, it may be that in the published examples where DPEs are observed, but corresponding PoP were not (as in Ariga and Kawahara, 2004), it may be due to a feature asymmetry in the category pair used for stimuli in that experiment, where only one of the two features in the pair systematically produced a pop-out^3^. Of course, this is but one possibility. There may very well be others, perhaps related to other (not low-level) differences with the stimuli used.

### ATTENTION DECISION-MAKING TASKS

Recently, Tseng et al. (2014) proposed that pop-out oddball tasks can be understood as attention decision-making tasks. Let us take the example of a color oddball task, with red and green color categories. The authors proposed that it is precisely because participants do not know which color is associated with target status (i.e., which color defines the oddball) and which with distractor status, that priming arises. On every trial, there is the need for a decision, and this decision is primed by recent trials. Shifting the emphasis away from perceptual processing, the authors thus modeled inter-trial priming as one does simple decisions (like two-alternative forced choice tasks), with one difference. The goal of the decision-making stage is not to arrive at an overt decision (which button to push), but rather to arrive at a covert decision: which color, in the current display, is associated with target status. Once a decision is reached, selection ensues and eventually, a response to the item is overtly measured. Note that the decision was not “which item (of the three items in the display) is the target,” but rather, the decisions pertained to the role of the visual categories that defined the oddball task. This attention decision-making stage is therefore in charge of mapping the two colors in the display (red and green) to the two roles in the task (target and distractor). Once that is achieved (say, red is found to be associated with target status), orienting to the corresponding item in the display is easy, given the conspicuity of the oddball. Within this framework, inter-trial effects arise as biases to maintain the same target/distractor to colors mapping as those established on preceding trials. That is, the inter-trial effects are best modeled as changes to decision boundaries. For instance, if on trial N-1, green was associated with distractor status, on trial N the decision boundary to decide that green is associated with target status is heightened, delaying selection of targets that happen to be green on trial N.

From this attention-decision making perspective, the current results suggest that the “decisions” made by the attention system are different on target-present and target-absent trials. This cannot be easily determined when only two features are used in the search task and both produce pop-out effects [as in ((s))Tseng et al. (2014) study and many of the early PoP studies]. Proposing that attentional decisions are different on target-present and target-absent trials is not a new concept. In fact, much of the literature on visual search has always treated differently these two types of trials, where special “termination” rules are said to apply to target-absent trials, simply because on those trials no single stimulus compels an action, and therefore the end of the trial (e.g., Wolfe, 1994; Wolfe and Van Wert, 2010). All that being said, we want to be clear that what we propose is not that the distractor tags are implemented in different ways in target-present and target-absent trials. The implementation of those tags is likely identical (see Tseng et al., 2014). But rather, what we argue is that the manner in which one arrives at those tags is different in these two scenarios.

Incidentally, from this attentional-decision making perspective, it is easy to make sense of a curious result in the PoP literature: some experiments have measured the magnitude of the inter-trial effects with varying set sizes and found that both RTs and the magnitude of the inter-trial effect decrease with increasing display set size (e.g., Bravo and Nakayama, 1992; Meeter and Olivers, 2006). This makes sense: the more items in the display there are, the faster a decision can be made regarding which color is associated with distractor status on that trial (the more distractors, the more evidence there is on the display regarding their color being the color associated with distractor status). This faster distractor-decision time produces a reduction in overall RT, as well as reduces the room to observe effects from recent status history.

Understanding inter-trial priming as a decision-making process also allows us to more easily understand the presence of analogous effects in temporal search tasks, where items are presented one at a time, rather than all at once, as in traditional spatial search tasks. In Lleras et al. (2009b), the authors presented participants with RSVP streams of colored letters and asked them to report the case (upper or lower) of the oddball colored letter. They found that selecting a color oddball in the stream was extremely difficult, if that color had been associated with distractor status on the preceding trial. This effect was only evident when the oddball appeared early in the stream, evidence that as the stream progressed, participants were actively changing the target-distractor assignments to the appropriate colors on the trial. As with spatial search tasks, detecting the presence of a color oddball produced no inter-trial effects. Once again, those results suggest that the decision to orient to (or select in time) one item amongst others is where the priming effect is taking place. Finally, in Levinthal and Lleras (2008b, also reported in Lleras et al., 2009a; see also Yashar and Lamy, 2010 for converging evidence with PoP), the authors showed that the target-distractor “tags” are independent of the search task in which they are created. The authors inter-mixed RSVP trials with spatial search trials and showed that inter-trial effects were observed to equal extents when participants completed two trials of the same type of search (spatial search followed by spatial search or RSVP search followed by RSVP search) than when participants switched tasks across trials (spatial search followed by RSVP search of RSVP search followed by spatial search). These findings are important because (a) they suggest a common, high-level locus to the target/distractor decision process; (b) they further suggest that these inter-trial effects are unrelated to modulations of the oddball’s conspicuity; and (c) they demonstrate a certain degree of commonality between the mechanisms in charge of selecting information in time with those selecting information in space.

### RELATION TO OTHER FORMS OF INTER-TRIAL PRIMING

In this paper, we have proposed a possible reason for the asymmetry that exists between two forms of inter-trial priming: PoP and the DPE. We proposed that this asymmetry is related to the fact that in order to observe PoP, the target needs to pop-out from the search display, whereas this is not a requirement for the DPE. That said, we are not claiming that target-centered inter-trial priming can only occur when the target pops-out. Below, we review other forms of inter-trial priming and we discuss how they relate to our current findings.

In the so-called dimension-priming paradigm, first reported by Müller et al. (1995, see also Found and Müller, 1996; Olivers and Meeter, 2006), the target is defined as an oddball in one of several possible feature dimensions. For instance, on trial N, the target might be a color oddball, whereas on trial N+1, the target could be an orientation oddball. In these types of tasks, participants do not know *a priori* which dimension of the display to scrutinize on any given trial. As noted by Olivers and Meeter (2006) the pattern of results obtained in these circumstances is somewhat different from the one observed in cases where there is no dimension uncertainty, i.e., when participants know *a priori* what dimension of the display defines the presence of a target, as with PoP and the DPE. When there is dimension uncertainty, priming is often stronger and more robust in present/absent tasks (i.e., it is easier to notice the presence of the oddball in the display if the oddball occurs along the same dimension on consecutive trials) than on compound task (reporting which color or orientation was the oddball on a given trial). In contrast, when there is dimension certainty the opposite pattern is observed: inter-trial priming occurs when participants are asked to report specifics about the target oddball (e.g., which side of the color is missing?) but no inter-trial priming is observed when participants are asked to report whether or not there is an oddball in the display (Lleras et al., 2008).

Müller et al. (1995) [as well as Olivers and Meeter (2006)] argued that in cases of dimension uncertainty the precise feature repetition is less important because the system is set up at a higher level to be sensitive to the larger change (dimension change) than to the specific one (which feature within a dimension). There is also a large literature showing that feature-based pop-out depends on top-down goals (i.e., knowing which dimension requires examination/focus). For instance, in the context of PoP, Fecteau (2007) found that in the typical PoP task of Nakayama, there was no priming for the unattended dimension. Our lab has shown this as well in the DPE (Levinthal and Lleras, 2008a). So, it appears that when there is dimensional uncertainty (i.e., when observers donot know which dimension of the stimulus will contain the target), detecting a repetition vs. alternation of dimension across trials is what is most important because the dimension sets for that trial the goal of focused attention. Notice too that, in the case of dimension uncertainty, biasing attention toward a specific feature repetition (say, red) affords little advantage to the viewer if there is no guarantee whatsoever that red (or even color in general) will produce a contrast on the following trial (because the next target could be defined as an orientation singleton). In contrast, when dimensions are certain, there is a goal for focused attention and there are “savings” to leave the biases in place from one trial to the next (Tseng et al., 2014). In sum, it is likely that priming under dimension uncertainty behaves quite in a different way that priming under featural uncertainty (but dimension certainty). It would be interesting to test with modeling whether dimensional priming can also be understood as a priming within a decision-making framework, with the goal being determining which dimension (not which specific feature) ought to be inspected on a trial-by-trial basis.

There is, of course, different levels of processing that may benefit from repetitions which would lead to inter-trial priming. This was best discussed by Ásgeirsson and Kristjánsson (2011), who showed that sometimes priming in visual search may arise from retrieval of episodic memories (or traces) of a preceding trial (see also Huang et al., 2004). Simply put, the current trial context facilitates retrieval of that episodic memory (when the context match across trials is high) and this in turn facilitates the latter stages of perceptual processing of the target object as a whole (see also Kristjánsson and Campana, 2010; Lamy et al., 2010). This form of priming has different characteristics than feature-based inter-trial priming. For example, feature-based priming applies to one specific feature (the attended feature) and does not extend to the unattended feature (see Fecteau, 2007; Levinthal and Lleras, 2008a; or only modestly, see Michal et al., submitted). Further, the benefit extends across the whole field to all objects who share that feature. Episodic priming is also mainly observed in difficult/inefficient search tasks, whereas feature-based priming seems to require efficient search (as suggested by the current results).

## CONCLUSION

The inter-trial effects known as Priming of Pop-out and the Distractor Preview Effect are, at face value, quite similar: they reflect the effects that switching or repeating target/distractor roles across trials can have on performance. In spite of this descriptive similarity, there is a deep difference between the two. The current results suggest that the manner in which the attention system arrives at those tags, and more specifically, at the distractor tag, is fundamentally different across the two phenomena. Assigning a visual category the status of distractor because attention easily detected the presence of a target (belonging to a separate category) does not appear to have the same effects (or to be processed neurally the same way) as assigning a status of distractor to a category because attention was “unable” to find a visual category that could be tagged as target. Additionally, the current study demonstrated the existence of PoP and DPE with fairly complex visual categories (numbers and letters) which have similar low-level characteristics, isolating the locus of the difference between these effects at a relatively high-level of processing. Finally, the study proposed one possible reason why DPEs may be observable with category pairs that do not allow for a PoP to emerge: it appears that PoP is only observable when both categories are capable of producing a pop-out effect, whereas for the DPE, having just one category that pops-out seems sufficient.

## Conflict of Interest Statement

The authors declare that the research was conducted in the absence of any commercial or financial relationships that could be construed as a potential conflict of interest.

## Footnotes

1 In the current article, we will be concerned with inter-trial priming where there is complete certainty as to what visual dimension or category will define the target in the visual search task. There exists a different type of inter-trial priming where there is uncertainty in that regard, the so-called dimension priming. In those cases, participants do not know ahead of a trial which feature dimension they should monitor for the presence of a target: the target can be an oddball by color in one trial, and by orientation the next. We discuss this further in the General Discussion.

2 To be clear, the DPE is not dependent on whether participants execute a response or not on target-absent trials (e.g., Lleras et al., 2008). What we emphasize here is that, on target-absent trials, attention does not get to “go” toward a target, and that’s what attention is trying to do on each trial.

3 Wan and Lleras (2010) published evidence that no DPE is found in inefficient search tasks when neither category pops-out (average slope of 22 ms/item).
